# A phase I study of enfortumab vedotin in Japanese patients with locally advanced or metastatic urothelial carcinoma

**DOI:** 10.1007/s10637-019-00844-x

**Published:** 2019-08-14

**Authors:** Shunji Takahashi, Motohide Uemura, Tomokazu Kimura, Yoshihide Kawasaki, Atsushi Takamoto, Akito Yamaguchi, Amal Melhem-Bertrandt, Elaina M. Gartner, Takashi Inoue, Rio Akazawa, Takeshi Kadokura, Toshiki Tanikawa

**Affiliations:** 1 Department of Medical Oncology, The Cancer Institute Hospital of Japanese Foundation for Cancer Research, Tokyo, Japan; 2 Osaka University Hospital, Osaka, Japan; 3 University of Tsukuba Hospital, Tsukuba, Japan; 4 Tohoku University Hospital, Sendai, Japan; 5 Okayama University Hospital, Okayama, Japan; 6 Harasanshin Hospital, Fukuoka, Japan; 7 Astellas Pharma Global Development, Northbrook, IL USA; 8 Seattle Genetics, Seattle, WA USA; 9 Astellas Pharma, Inc., Tokyo, Japan; 10 Niigata Cancer Center Hospital, Niigata, Japan

**Keywords:** Immunoconjugates, Japan, Nectins, Neoplasms, Urothelium

## Abstract

Locally advanced or metastatic urothelial cancer is an aggressive form of cancer with high recurrence rates and low survival. Nectin-4 is a cell adhesion molecule commonly expressed in several tumors, including high expression in urothelial cancer. Enfortumab vedotin is an antibody–drug conjugate composed of an anti-Nectin-4 humanized monoclonal antibody linked to the microtubule disrupting agent, monomethyl auristatin E. In this phase I study (NCT03070990), Japanese patients with locally advanced/metastatic urothelial cancer treated with prior chemotherapy, or ineligible for cisplatin, were randomized 1:1 to receive 1.0 mg/kg (*Arm A*) or 1.25 mg/kg (*Arm B*) enfortumab vedotin on Days 1, 8, and 15 of each 28-day cycle. Assessing the pharmacokinetic and safety/tolerability profiles of enfortumab vedotin were primary objectives; investigator-assessed antitumor activity (RECIST v1.1) was a secondary objective. Seventeen patients (*n* = 9, *Arm A*; *n* = 8, *Arm B*) received treatment. Pharmacokinetic data suggest a dose-dependent increase in enfortumab vedotin maximum concentration and area under the concentration–time curve at Day 7. Enfortumab vedotin was well tolerated across both doses. Dysgeusia and alopecia (*n* = 9 each) were the most common treatment-related adverse events. Regardless of attribution, grade ≥ 3 adverse events occurring in ≥2 patients were anemia and hypertension (*n* = 2 each). One patient achieved a confirmed complete response (*Arm A*) and five achieved confirmed partial responses (*n* = 3, *Arm A*; n = 2, *Arm B*). Objective response and disease control rates were 35.3% and 76.5%, respectively. In Japanese patients with locally advanced/metastatic urothelial cancer, enfortumab vedotin is well tolerated with preliminary antitumor activity and a pharmacokinetic profile consistent with prior reports.

**Electronic supplementary material:**

The online version of this article (10.1007/s10637-019-00844-x) contains supplementary material, which is available to authorized users.

## Introduction

Globally and historically advanced stages of urothelial carcinoma have been difficult to treat. In Japan, the 2012 incidence rate for locally advanced or metastatic urothelial cancer (UC) was estimated as 2.8 per 100,000 patients [1]. Chemotherapy combinations of cisplatin plus gemcitabine, or cisplatin plus methotrexate, vinblastine, and doxorubicin, are the established, standard first-line regimens for the treatment of locally advanced or metastatic UC throughout the United States, Europe, Canada, and Japan [2, 3]. Unfortunately, a large number of patients are cisplatin-ineligible at the time of diagnosis, most commonly because of renal insufficiency. As a result, carboplatin with gemcitabine is frequently an alternative regimen for these patients [4]. While the initial response rates range from 50% to 70%, few patients have durable responses and many patients become resistant to the initial treatment, and for those patients who fail on first-line chemotherapy, the 5-year survival rate is low (5%) [1]. Pembrolizumab, an antibody against programmed death-1 receptor (PD-1), was recently approved in Japan for the treatment of metastatic UC. This approval was based on results from the KEYNOTE-045 study where the objective response rate (ORR) was 21% and overall survival (OS) was 10.3 months [5]. Other immune checkpoint inhibitors targeting PD-1/programmed death ligand-1 (PD-L1) have been approved outside of Japan for the second-line treatment of patients with metastatic UC. However, there are no other non-platinum-based therapies approved for second-line treatment in Japan at this time, and as such, there is a clear need to develop new therapies for metastatic UC.

Nectin-4 is a type I transmembrane cell adhesion protein that has been found to be highly expressed in a number of epithelial cancers, most notably in UC with high expression [6]. In normal tissue, Nectin-4 expression is moderate to weak and is mainly found in the epithelium of the bladder, skin, salivary gland (ducts), gastrointestinal tract, and breast ducts [6–9]. Both the expression pattern and levels of Nectin-4 on UC tumors suggest it may be an attractive target for a therapeutic antibody–drug conjugate (ADC) in this disease [10].

The goal of ADC cancer therapy is to improve the therapeutic index and target the tumor, thus minimizing exposure to normal tissue. Enfortumab vedotin (EV) is a novel, fully humanized, monoclonal ADC that delivers the microtubule-disrupting agent, monomethyl auristatin E (MMAE), to cells expressing Nectin-4 [6]. Enfortumab vedotin selectively binds cell-surface Nectin-4 with high affinity. Following binding to Nectin-4, EV is internalized and undergoes proteolytic cleavage and intracellular release of MMAE. The free MMAE disrupts tubulin polymerization and leads to mitotic arrest [6].

In a large phase I dose-escalation/dose-expansion study conducted in North America (EV-101), EV was found to be generally well tolerated in adult patients who had undergone at least one prior line of chemotherapy or were ineligible for cisplatin-based chemotherapy; nearly half of the patients with metastatic UC were from a prospectively enrolled cohort of patients previously treated with anti-PD-1 or PD-L1 (PD-[L]1) therapy. Fatigue, alopecia, nausea, and decreased appetite were commonly reported treatment-related adverse events (TRAEs) and the majority of adverse events (AEs) considered related to EV were mild to moderate in severity. This study also estimated the recommended phase II dose (RP2D) of EV as 1.25 mg/kg on Days 1, 8, and 15 of each 28-day cycle; the maximum tolerated dose was not reached. Across the 112 patients with metastatic UC treated with EV 1.25 mg/kg, the observed response rate and durability were promising with an investigator-assessed confirmed ORR of 43% and a median duration of response (DoR) of 7.4 months [11].

To address the unmet need of Japanese patients with locally advanced or metastatic UC, a phase I open-label study in Japan was designed to evaluate the safety/tolerability and pharmacokinetic (PK) profiles of EV, as well as its antitumor activity and immunogenicity (as defined by the incidence of anti-drug antibodies [ADAs]).

## Materials and methods

### Patients

Adult Japanese patients (≥20 years) with histologically confirmed, locally advanced or metastatic transitional cell carcinoma of the urothelium (ie, cancer of the bladder, renal pelvis, ureter, or urethra), or UC with squamous differentiation or mixed cell types and an Eastern Cooperative Oncology Group (ECOG) performance status of 0 or 1, were eligible. Patients must have failed at least one prior chemotherapy regimen for advanced disease unless considered unfit for cisplatin-based chemotherapy. Patients were excluded if they had pre-existing sensory or motor neuropathy (grade ≥ 2) or immunotherapy-related AEs requiring high doses of systemic steroids (≥20 mg/day equivalent of prednisone), uncontrolled nervous system metastasis requiring active treatment, or significant cardiovascular disease. Patients with the following were also excluded: a known history of a positive test for human immunodeficiency virus infection; uncontrolled diabetes mellitus or diabetic neuropathy within 3 months of the first dose of study drug; thromboembolic events and/or bleeding disorders (eg, stroke, DVT, or PE) ≤14 days prior to the first dose of the study drug; ocular conditions, such as infection or corneal ulcer (keratitis); monocularity; history of corneal transplantation; uncontrolled glaucoma (topical medications allowed), or uncontrolled or evolving retinopathy; wet macular degeneration; uveitis; papilledema; or optic disc disorder.

Patients were also required to submit a tumor tissue sample for Nectin-4 analysis at a central laboratory. Nectin-4 expression levels were determined by image analysis of Nectin-4 immunohistochemistry results of tumor biopsy samples. Immunohistochemistry staining results were scored with the H-score method (H-score = [(percentage of strong positive tumor cells) X 3] + [(percentage of moderate positive tumor cells) X 2] + [percentage of weak positive tumor cells) X 1]) and ranged from 0 to 300. Nectin-4-positive tumor cells were classified by a pathologist as strong, moderate, or weak depending on staining intensity.

### Study design and conduct

This open-label randomized study of EV was conducted in Japanese patients diagnosed with locally advanced or metastatic UC to assess the safety/tolerability, PK, and antitumor activity of EV. A total of 19 patients were randomly assigned 1:1 to one of two treatment arms (*Arm A* or *Arm B*). Patients in *Arm A* received EV at 1.0 mg/kg and patients in *Arm B* received EV at 1.25 mg/kg, which was the estimated RP2D in North American patients with metastatic UC.

All patients who met the eligibility criteria were randomized according to the randomization schedules through Interactive Response Technology (IRT) and site personnel dispensed the treatment according to the IRT system’s assignment. Patients assigned to *Arm A* were allowed to dose escalate to 1.25 mg/kg at the investigator’s discretion and if there were no significant toxicities during the first cycle of therapy. Patients continued treatment until disease progression, clinically significant toxicity, investigator decision, or informed consent withdrawal.

### Treatment

All patients received a 30-min IV infusion of EV on Days 1, 8, and 15 of each 28-day cycle; patients continued treatment until one of the discontinuation criteria was met (Online Resource 1). During Cycle 1, patients were administered EV in the inpatient setting. Enfortumab vedotin was administered at mg/kg doses based on the subject’s actual body weight at baseline (ie, Cycle 1 Day 1) and doses did not need to change unless the subject’s weight changed by ≥10% from their baseline weight or the dose adjustment criteria were met. Both tested doses (1.0 and 1.25 mg/kg) were anticipated to be safe and active in Japanese patients with locally advanced or metastatic UC.

### Assessments

Pharmacokinetic samples were collected on Cycle 1 Day 1 predose, end of infusion (EOI), 30 min post-EOI, and 2, 4, 24 (Day 2), 48 (Day 3), and 72 (Day 4) hours postdose. On Cycle 1 Day 8, PK samples were collected predose and at the EOI. On Cycle 1 Day 15, PK samples were collected predose, EOI, 30 min post-EOI, and 2, 4, 24 (Day 16), 48 (Day 17), 72 (Day 18), and 168 (Day 22) hours postdose. On Cycle 2, PK samples were collected on Day 1 (predose) and at the end of infusion. An additional PK sample was collected predose on Day 1 of Cycles 3 and 4, predose on Day 1 of all even cycles thereafter, and at the safety follow-up visit. Blood samples for ADA analyses were also collected predose on Day 1 of Cycles 1, 2, 3, 4, and even cycles thereafter, as well as at the safety follow-up visit.

While all treatment-emergent and -related AEs were evaluated across the entire study, EV tolerability was specifically evaluated between Cycle 1 Day 1 and predose of Cycle 2. Enfortumab vedotin was considered tolerable, unless ≥3 patients per arm experienced any of the following TRAEs included in the *Safety Evaluation Criteria*: grade 4 neutropenia lasting more than 7 days; grade 4 febrile neutropenia, grade 4 thrombocytopenia, or grade 3 thrombocytopenia with clinically significant bleeding requiring platelet transfusion; grade 4 anemia or grade 3 anemia requiring a red blood cell transfusion; grade ≥ 3 non-hematological laboratory abnormalities with clinical consequences that do not resolve within 7 days; nonlaboratory grade ≥ 3 AEs, with the exception of grade 3 rash that is not a serious AE or does not result in discontinuation of study drug, and grade 3 infusion reaction that resolves within 24 h; or a patient who did not receive all three EV doses during Cycle 1 for drug-related AEs not specified above. Adverse events clearly related to disease, pre-existing conditions, and environmental factors were excluded.

Complete eye exams were performed on Cycle 1 Day 22, Cycle 3 Day 1, and Day 1 of every odd cycle. If three consecutive eye exams did not show significant findings consistent with a treatment-related ocular change, ophthalmic exams were only conducted at the onset of any eye symptom(s) during study drug treatment.

Electrocardiogram (ECGs) data were collected on Days 1, 3, 15, and 17 in Cycle 1. An ECG was also collected at the safety follow-up visit. Routine 12-lead ECGs were performed after the patient had been in a supine position for at least 5 min. All ECGs were collected in triplicate at 2-min intervals and were centrally analyzed.

Antitumor activity was assessed via radiologic imaging (CT scans/MRI), which was performed at baseline and every 8 weeks (± 1 week) thereafter. The modality used at baseline would be used throughout the study for imaging assessment; both response and progression were assessed locally according to RECIST v1.1.

### Statistical analyses

Safety/tolerability of EV was a primary endpoint in this study and was evaluated in all patients who were randomized and received ≥1 dose of EV. A treatment-emergent AE (TEAE) was defined as an AE observed after starting administration, and up to 28 days after last administration, of the study drug. A TRAE was defined as any TEAE with at least a possible relationship to study treatment as assessed by the investigator or with missing assessment of the causal relationship.

The EV PK profile was also a primary endpoint. Drug concentrations and standard PK parameters (eg, AUC [area under the concentration–time curve], C_max_ [maximal concentration], T_max_ [time of maximal concentration]) were determined from patients in the pharmacokinetic analysis set (PKAS), which consisted of all patients who were randomized and received ≥1 dose of study drug, for whom sufficient concentration data were available to facilitate derivation of at least one PK parameter, and for whom the time of dosing on the day of sampling was known. Data from the PKAS were summarized by dose level using descriptive statistics and the time course of drug concentrations were plotted.

Antitumor activity was a secondary endpoint and evaluated in all patients who were randomized, received ≥1 dose of study drug, and were evaluated for at least one baseline efficacy endpoint. Clinical response, including ORR and disease control rate (DCR), was assessed based on RECIST v1.1. Duration of response and progression-free survival (PFS) were estimated using Kaplan-Meier analysis with defined censoring rules (Online Resource 2). Time-to-response (TTR) was defined as the time from the start of the study treatment until first response (confirmed complete response [CR] or partial response [PR]); TTR was calculated for all objective responders.

### Study oversight

A Data and Safety Monitoring Board (DSMB) provided trial oversight. This study was designed by the study sponsor in collaboration with the investigators and was conducted in accordance with the Declaration of Helsinski and Good Clinical Practice guidelines, principles of informed consent, and requirements of the public registration of clinical trials (ClinicalTrials.gov Identifier, NCT03070990). In addition, the research protocol was approved by each site’s Institutional Review Board/Independent Ethics Committee/Research Ethics Board and informed consent was obtained from all study participants at the time of enrollment.

### Role of the funding source

Astellas Pharma, Inc. and Seattle Genetics, Inc. provided funding for this trial and were involved in the development of the study protocol, and in data collection, analysis, and interpretation. Editorial and writing assistance during the development of the manuscript was supported by both Astellas Pharma, Inc. and Seattle Genetics, Inc. The corresponding author had full access to all of the data in the study and final responsibility for the decision to submit the paper for publication.

### Data sharing statement

Studies conducted with product indications or formulations that remain in development are assessed after study completion to determine if Individual Participant Data can be shared. The plan to share Individual Participant Data is based on the status of product approval or termination of the compound, in addition to other study-specific criteria described on www.clinicalstudydatarequest.com under “Sponsor Specific Details for Astellas.”

## Results

### Study disposition

A total of 24 Japanese patients with locally advanced or metastatic UC gave informed consent to participate in the study and prior to randomization five patients failed screening. Nineteen patients participated in the study and were randomized to treatment (*Arm A*, *n* = 10; *Arm B*, *n* = 9), but one patient in each arm did not receive any study drug. Across the 17 patients who received at least one dose of EV, the median patient age was 68 years (range: 57–82). Most patients were male (*n* = 15; 88.9%) with a baseline ECOG performance score of 0 (*n* = 13; 76.5%) and a mean baseline estimated glomerular filtration rate (eGFR) of 61.89 ± 12.64 mL/min/1.73 m^2^. All patients had prior cisplatin-based treatment; one patient in *Arm A* was previously treated with an immune checkpoint inhibitor. Bladder was the site of the primary tumor in ~70% of patients and eight (47.1%) patients had metastasis to visceral tissue (ie, liver, lung, and adrenal gland). The median immunohistochemistry H-score for tissue Nectin-4 expression was 290 (range: 6, 300). There were no remarkable differences in demographic characteristics between *Arms A* and *B* (Table 1).

**Table 1:** Demographic and baseline characteristics of Japanese patients with locally advanced or metastatic UC

	*Arm A* 1.0 mg/kg EV (*n* = 9)	*Arm B* 1.25 mg/kg EV (*n* = 8)	Total (*N* = 17)
Sex,* n* (%)			
Male	8 (88.9)	7 (87.5)	15 (88.2)
Female	1 (11.1)	1 (12.5)	2 (11.8)
Median age, years (range)	67.0 (61, 82)	67.5 (57, 78)	67.0 (57, 82)
Median tissue Nectin-4 expression IHC H-score (range)	295.0 (190, 300)	262.5 (6, 300)	290 (6, 300)
Baseline ECOG performance status, *n* (%)			
0	7 (77.8)	6 (75.0)	13 (76.5)
1	2 (22.2)	2 (25.0)	4 (32.5)
Mean baseline eGFR, mL/min/1.73 m^2^ (SD)	59.1 (10.6)	65.0 (14.7)	61.9 (12.6)
Site of primary tumor, *n* (%)			
Bladder	6 (66.7)	6 (75.0)	12 (70.6)
Renal pelvis	1 (11.1)	2 (25.0)	3 (17.6)
Ureter	2 (22.2)	0	2 (11.8)
Site of metastasis at baseline, *n* (%)			
Bone	3 (33.3)	3 (37.5)	6 (35.3)
Liver*	2 (22.2)	0	2 (11.8)
Lung*	2 (22.2)	4 (50.0)	6 (35.3)
Adrenal gland*	1 (11.1)	1 (12.5)	2 (11.8)
Brain	0	1 (12.5)	1 (5.9)
Other	8 (88.9)	5 (62.5)	13 (76.5)

*Considered visceral metastatic sites

Abbreviations: ECOG, Eastern Cooperative Oncology Group; eGFR, estimated glomerular filtration rate; H-score, histoscore; IHC, immunohistochemistry; SD, standard deviation; UC, urothelial cancer

### Pharmacokinetic profile of EV

**Fig. 1:**
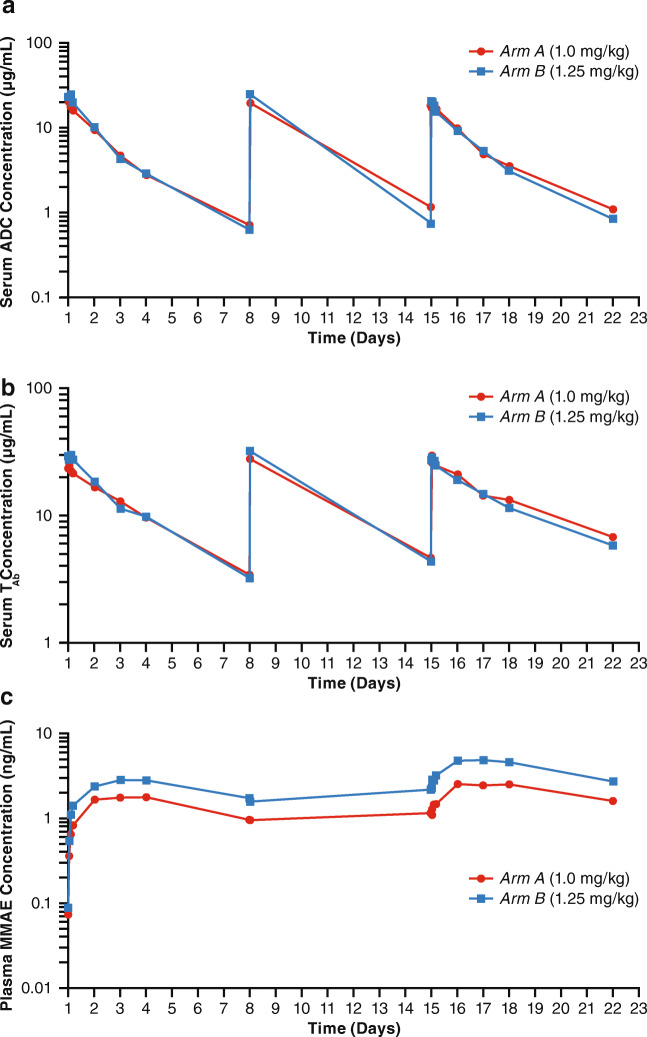
**Mean Serum Concentration Profile at Cycle 1 of (a) ADC, (b) T**_**Ab**_**, (c), and MMAE (Semi-Log Scale Plot).** Abbreviations: ADC, antibody–drug conjugate; MMAE, monomethyl auristatin E; T_Ab,_ total antibody

The mean serum concentration profiles of ADC, total antibody (T_Ab_), and mean plasma MMAE in Cycle 1 are presented in Fig. 1a–c; PK parameters for ADC, T_Ab_, and MMAE on Days 1 and 15 of Cycle 1 are presented in Table 2. Following the first dose of EV, intact ADC exposure (AUC_7d_) and observed C_max_ were generally increased with increased dose. After the end of infusion, serum ADC concentrations appeared to decrease exponentially and minimal intra-cycle accumulation, as assessed by the mean accumulation ratio (R_ac_), was observed. Total antibody concentrations were generally higher than the corresponding intact ADC concentrations and exposure to T_Ab_ appeared to increase in a dose-dependent manner. Plasma MMAE concentrations appeared to increase following infusion and reach maximum concentrations at 2 to 3 days after infusion.

**Table 2:** Pharmacokinetic parameters for intact antibody–drug conjugate, total antibody, and monomethyl auristatin E

	*Arm A* 1.0 mg/kg EV		*Arm B* 1.25 mg/kg EV	
	Day 1	Day 15	Day 1	Day 15
ADC				
AUC_7d_	29.2 (4.4)	32.9 (7.0)	30.6 (5.2)	30.8 (4.4)
C_max_	20.3 (4.2)	20.8 (6.0)	25.3 (7.1)	20.9 (1.5)
R_ac_ (C_max_)	NA	1.1 (0.19)	NA	1.1 (0.08)
T_Ab_				
AUC_7d_	67.8 (9.4)	92.7 (19.0)	69.2 (9.8)	84.9 (7.1)
C_max_	24.7 (3.6)	31.2 (8.2)	30.2 (6.3)	29.7 (3.8)
R_ac_ (C_max_)	NA	1.4 (0.38)	NA	1.2 (0.13)
MMAE				
AUC_7d_	5.5 (−)	10.7 (−)	8.9 (−)	63.7 (−)
C_max_	1.9 (0.9)	2.7 (1.3)	2.9 (2.6)	5.0 (4.3)
t_max_ (hr)*	48.5 (24, 73)	48.5 (24, 71)	49.7 (24, 72.2)	46.6 (23.4, 47.3)
R_ac_ (C_max_)	NA	1.7 (0.5)	NA	1.6 (0.28)

Abbreviations: ADC, antibody-drug conjugate; MMAE, monomethyl auristatin E; NA, not available; T_Ab,_ total antibody

*Median (min, max)

### Safety and tolerability of EV

While tolerability of EV was evaluated across the course of the study, the *Safety Evaluation Criteria* was evaluated from Cycle 1 Day 1 until predose of Cycle 2 Day 1. During this assessment, EV-related AEs listed in the *Safety Evaluation Criteria* were reported in two of nine patients (anemia and pyrexia, *n* = 1 each) in *Arm A* and three EV-related AEs (bacterial pneumonia, erythematous rash, and hypertension) were reported in two of eight patients in *Arm B*. Based on safety and PK data, the DSMB considered both doses (1.0 or 1.25 mg/kg) tolerable.

Throughout the conduct of the study, all patients reported experiencing at least one AE. Enfortumab vedotin-related AEs were reported in 15 of the 17 patients (*n* = 9, *Arm A*; *n* = 6, *Arm B*); treatment-related AEs occurring in ≥3 patients in either treatment arm are presented in Table 3. The most commonly reported AEs possibly related to EV (occurring in ≥30% of patients) were dysgeusia and alopecia (52.9%, *n* = 9 each), dry skin and pruritus (47.1%, *n* = 8 each), anemia and decreased appetite (41.2%, *n* = 7 each), and pyrexia (35.3%, *n *= 6). As Nectin-4 is present in normal human skin and neuropathy has been previously reported with microtubule inhibitors like MMAE, treatment-related rash and peripheral neuropathy were expected AEs. Regardless of attribution, grade ≥ 3 AEs were reported in 10 of the 17 patients (*n* = 5, *Arm A*; *n* = 5, *Arm B*). Anemia (*n* = 3) and hypertension (*n* = 2) were the only grade ≥ 3 AEs reported in ≥2 patients.

**Table 3:** Treatment-related adverse events (all grades) occurring in ≥3 patients in either treatment arm

	*Arm A* 1.0 mg/kg EV (*n* = 9)	*Arm B* 1.25 mg/kg EV (*n* = 8)	Total (*N* = 17)
Alopecia	6 (66.7)	3 (37.5)	9 (52.9)
Dysgeusia	5 (55.6)	4 (50.0)	9 (52.9)
Dry skin	5 (55.6)	3 (37.5)	8 (47.1)
Pruritus	5 (55.6)	3 (37.5)	8 (47.1)
Anemia	6 (66.7)	1 (12.5)	7 (41.2)
Decreased appetite	4 (44.4)	3 (37.5)	7 (41.2)
Pyrexia	3 (33.3)	3 (37.5)	6 (35.3)
Decreased neutrophil count	4 (44.4)	1 (12.5)	5 (29.4)
Increased aspartate aminotransferase	4 (44.4)	1 (12.5)	5 (29.4)
Malaise	3 (33.3)	2 (25.0)	5 (29.4)
Peripheral sensory neuropathy	5 (55.6)	0	5 (29.4)
Rash	3 (33.3)	2 (25.0)	5 (29.4)
Decreased weight	3 (33.3)	1 (12.5)	4 (23.5)
Decreased white blood cell count	4 (44.4)	0	4 (23.5)
Diarrhea	3 (33.3)	1 (12.5)	4 (23.5)
Fatigue	4 (44.4)	0	4 (23.5)

Data presented as* n* (%)

Two patients in *Arm A* each experienced one TRAE that lead to withdrawal (peripheral sensory neuropathy, *n* = 1; abnormal hepatic function, * n* = 1); in *Arm B*, one patient experienced two TRAEs (pneumonia and rash) that led to withdrawal. Serious AEs were reported in seven of the 17 patients (*n* = 4, *Arm A*; *n *= 3, *Arm B*); one patient experienced a fatal AE (disease progression), unrelated to EV, 14 days after the last dose.

No clinically meaningful changes in vital signs, clinical laboratory values, or substantial shifts in urinalysis parameters were found. Increased alanine aminotransferase and/or aspartate aminotransferase >3 x upper level of normal was reported in two patients in *Arm A*; however, neither patient met Hy’s law criteria for drug-induced liver injury.

Generally, no clinically meaningful mean changes from baseline were found in the ECG parameters. Clinically significant ECG abnormalities were observed postdose in three patients in *Arm A.* In one patient, abnormalities were reported at both screening and postdose time points; however, these were not reported as TEAEs. Abnormalities for the other two patients were observed only postdose and included a grade 1 TRAE of atrioventricular block first degree and right bundle branch block in one patient, and a grade 1 TRAE of atrioventricular block first degree in the other. The dose of EV in these patients was not changed on account of these ECG abnormalities. None of the patients had a QTcF interval > 450 msec or a change from baseline >60 msec. Two clinically significant ophthalmologic abnormalities, both of which were considered at least possibly related to treatment, were observed in one patient in *Arm A* at Cycle 5 Day 1 and in an unscheduled visit on Day 284 (dry eye and cataract). Two patients in *Arm B* had clinically significant cataracts. The cataracts were observed in one patient at screening, Cycle 1 Day 22, Cycle 3 Day 1, and Cycle 5 Day 1; in the second patient the cataracts were observed at Cycle 5 Day 1. The cataracts were not considered related to EV by the investigators in either patient. Study dose of EV was not changed on account of these ophthalmologic abnormalities.

### Immunogenicity of EV

Immunogenicity of EV was tested in all 17 patients. Of the 17 patients, 16 remained ADA-negative throughout treatment; however, one patient became transiently positive after exposure to EV (Cycle 2 Day 1), and resolved upon subsequent testing even after continued dosing with EV.

### Antitumor activity of enfortumab vedotin

Across all 17 patients, one patient (*Arm A)* achieved a confirmed CR, five (*n* = 3, *Arm A*; *n* = 2, *Arm B*) achieved a confirmed PR, and seven (*n* = 5, *Arm A*; *n* = 2, *Arm B*) patients had stable disease (Table 4). The ORR and DCR for the whole population were 35.3% and 76.5%, respectively. In *Arm A*, the confirmed ORR was 44.4% and DCR was 100%; one patient with stable disease had an unconfirmed PR. In *Arm B*, the confirmed ORR and DCR were 25% and 50%, respectively. Two patients in *Arm B* had progressive disease (PD) and two were not evaluable due to lack of a post-baseline assessment.

**Table 4:** Best confirmed overall response

	*Arm A* 1.0 mg/kg EV (*n* = 9)	*Arm B* 1.25 mg/kg EV (*n* = 8)	Total (*N* = 17)
Complete response (CR)	1 (11.1)	0	1 (5.9)
Partial response (PR)	3 (33.3)	2 (25.0)	5 (29.4)
Stable disease (SD)	5 (55.6)	2 (25.0)	7 (41.2)
Progressive disease (PD)	0	2 (25.0)	2 (11.8)
No post-baseline assessment	0	2 (25.0)	2 (11.8)
Objective response rate (ORR)	**4 (44.4)**	**2 (25.0)**	**6 (35.3)**
Disease control rate (DCR)	**9 (100.0)**	**4 (50.0)**	**13 (76.5)**

Data presented as *n* (%)

ORR = CR + PR; DCR = CR + PR + SD

CR/PR had to be confirmed by two scans a minimum of 28 days apart; the minimum duration for SD was 49 days

Bold font indicates composite endpoint

**Fig. 2:**
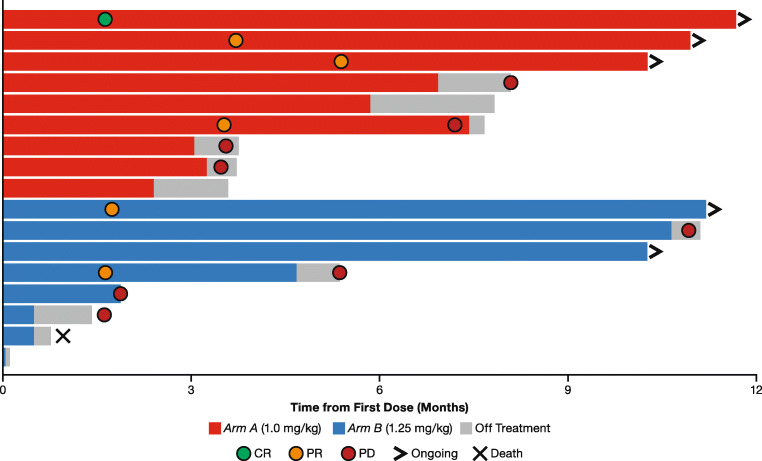
**Time to and Duration of Response.** Abbreviations: CR, complete response; PD, progressive disease; PR, partial response

**Fig. 3:**
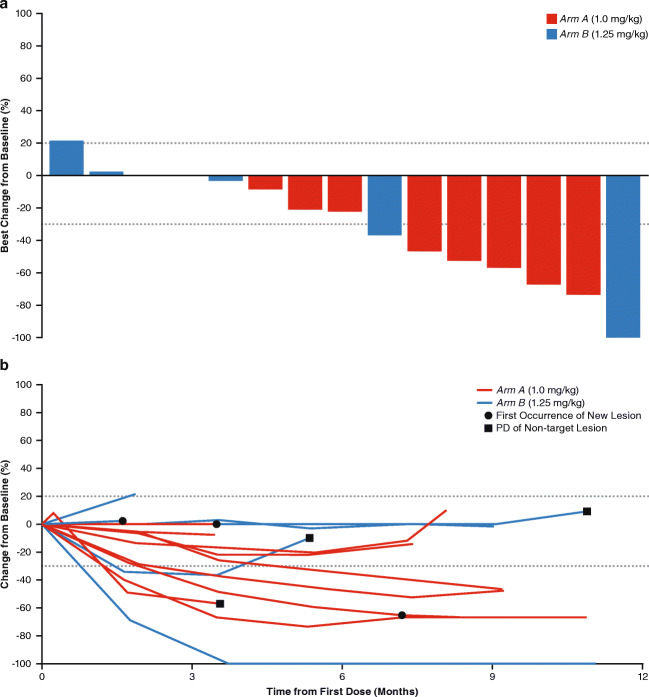
**Antitumor Effects of Enfortumab Vedotin in Patients With Metastatic UC (a) Change in Tumor Size From Baseline and (b) Percent Change in Tumor Size.** Abbreviations: PD, progressive disease; UC, urothelial cancer

Half of confirmed responses (CR, *n* = 1; PR, n = 2) occurred within the first 3 months and all occurred within 6 months (Fig. 2). After 9 months of treatment, three of the four responders in *Arm A* and the two responders in *Arm B* had ongoing responses. As of June 2018, median DoR could not be estimated due to the continued ongoing response; however, the response durations ranged from 3.7 to 9.3 months. The majority of patients with both baseline and at least one postbaseline measurement (*n* = 15) had tumor shrinkage (Fig. 3a), and tumor shrinkage was observed at the first disease assessment (Fig. 3b). Progression-free survival events occurred in a total of nine out of 17 patients across both treatment arms (*n* = 4, *Arm A*; *n* = 5, *Arm B*). From these events, the median PFS was estimated as 8.1 months (95% CI: 3.5, −).

## Discussion

The current therapeutic landscape for patients with locally advanced or metastatic UC in Japan lacks treatment options beyond the first-line setting that are both efficacious and tolerable. In this two-arm phase I study in Japanese patients with locally advanced or metastatic UC who had received prior chemotherapy or who were ineligible for cisplatin, single-agent EV, an ADC against Nectin-4, was safe, generally well tolerated with dose-proportional increases in exposure (AUC_7d_ and C_max_), and demonstrated antitumor activity. Furthermore, pre-screening of tumor biopsy samples in this study identified high levels of Nectin-4-positive UC cells. The high median H-score observed was consistent with results in the North American study, demonstrating that this target is relevant to Japanese patients with UC.

The PK profile of EV in Japanese patients was consistent with prior reports in Caucasian patients [11–13]. Mean exposures of ADC, T_Ab_, and MMAE generally increased with ascending dose and concentrations appeared to decrease multi-exponentially following the end of infusion, while minimal intra-cycle accumulation was observed for ADC. When comparing the PK profile of EV in Japanese patients to results from the phase I dose-escalation/dose-expansion study (EV-101) conducted in North America, no apparent differences in exposure were observed; thus, no dose adjustment is required for the Japanese population.

In Japanese patients, both doses of EV (1.0 and 1.25 mg/kg) were generally well tolerated with no significant differences in tolerability between doses; the safety/tolerability profile in this study was consistent with prior reports from the EV-101 study [11–13]. During Cycle 1, safety of each dose was assessed. Only two patients in each arm experienced ≥1 of the specific EV-related AEs; therefore, EV was considered tolerable. Across the study, the most commonly reported AEs were anemia, dysgeusia, and alopecia. The majority of AEs were mild to moderate in severity, with anemia being the most common grade ≥ 3 AE (*n* = 3/17). Peripheral neuropathy is a common AE particularly associated with MMAE conjugates due to the disruption of interphase microtubule function [14]. In this study, peripheral sensory neuropathy was observed in 29% of all patients; all peripheral neuropathy events occurred in *Arm A*, which may be a result of more patients in that arm remaining on treatment for a longer period of time. All events of peripheral sensory neuropathy were considered related to EV, and one led to treatment discontinuation. Expression of the therapeutic target on normal cells may result in on-target toxicities [15]. Toxicities associated with skin (eg, alopecia, dry skin, pruritus, and rash) were expected as Nectin-4 is normally expressed on human skin keratinocytes and appendages. Treatment-related rash was observed in 29% of all patients in *Arm A* and *Arm B*; one event of rash erythematosus led to treatment discontinuation. None of the fatal AEs were considered related to EV.

Clinical response to EV in Japanese patients with locally advanced or metastatic UC were encouraging. Consistent with the results reported in a North American metastatic UC population with similar inclusion criteria, the ORR (35.3%) and DCR (76.5%) rates were high, responses were durable, and activity was observed by 8 weeks after the first dose. At the time of data cutoff, median PFS was estimated as 8.1 months with eight patients (47%) who remained free from disease progression.

While promising, these data are limited by the small sample size. Furthermore, this study was conducted to assess the safety/tolerability and PK profile of EV in the Japanese population; this study was not designed as a comparison with other second-line therapies for advanced/metastatic UC. The safety/tolerability data from this study indicate that EV was well tolerated in Japanese patients and no new safety signals were observed. Based on the PK and safety data observed in this study, dosing that was established in EV-101 does not need to be adjusted for the Japanese patient population. The benefit-risk profile supports the continued evaluation of EV in Japanese patients with locally advanced or metastatic UC. Japanese patients previously treated with an anti-PD-(L)1 are being enrolled in two global studies: a single-arm, multicohort phase II study currently enrolling patients who are platinum naive and cisplatin-ineligible (EV-201; NCT03219333) and a randomized phase III trial of EV versus chemotherapy (docetaxel, paclitaxel, or vinflunine [EU only]) in patients with locally advanced or metastatic UC who were previously treated with an immune checkpoint inhibitor (EV-301; NCT03474107).

## Electronic supplementary material

Online Resource 1 (PDF 177 kb)

Online Resource 2 (PDF 236 kb)
