# Supplementary material for: A phase I study of enfortumab vedotin in Japanese patients with locally advanced or metastatic urothelial carcinoma
Source: Invest New Drugs. 2019 Aug 14;38(4):1056–66. doi: 10.1007/s10637-019-00844-x (PMC7340645; doi:10.1007/s10637-019-00844-x)
Supplement: Supplementary file 1 — (PDF 177 kb) [file 10637_2019_844_MOESM1_ESM.pdf]

Online Resource 1

A Phase I Study of Enfortumab Vedotin in Japanese Patients With Locally Advanced or Metastatic Urothelial Carcinoma

Investigational New Drugs

Shunji Takahashi[1], Motohide Uemura[2], Tomokazu Kimura[3], Yoshihide Kawasaki[4], Atsushi Takamoto[5], Akito Yamaguchi[6], Amal Melhem-Bertrandt[7], Elaina M. Gartner[8], Takashi Inoue[9], Rio Akazawa[9], Takeshi Kadokura[9], Toshiki Tanikawa[10]

[1]The Cancer Institute Hospital of Japanese Foundation for Cancer Research, Tokyo, Japan; [2]Osaka University Hospital, Osaka, Japan; [3]University of Tsukuba Hospital, Tsukuba, Japan; [4]Tohoku University Hospital, Sendai, Japan; [5]Okayama University Hospital, Okayama, Japan; [6]Harasanshin Hospital, Fukuoka, Japan; [7]Astellas Pharma Global Development, Northbrook, IL, USA; [8]Seattle Genetics, Seattle, WA, USA; [9]Astellas Pharma, Inc., Tokyo, Japan; [10]Niigata Cancer Center Hospital, Niigata, Japan

Corresponding author:

Shunji Takahashi, MD

E-mail: s.takahashi-chemotherapy@jfcr.or.jp

Additional Discontinuation Criteria

The following were discontinuation criteria from treatment for individual patients:

- Patient developed progressive disease (PD)
  - Progressive disease was defined as worsening of signs and symptoms that in the judgment of the investigator is indicative of progressive disease or according to the RECIST criteria (version 1.1)
- Patient required another systemic anticancer treatment for underlying or new cancer
- Patient developed unacceptable toxicity
- Pregnancy
- Investigator decides it is in the subject’s best interest to discontinue (eg, includes clinical progression)
- Patient declined further treatment or withdrew consent
- Patient was noncompliant with the protocol based on the investigator or medical monitor assessment
- Patient was lost to follow-up despite reasonable efforts by the investigator to locate the subject
- Death
