# Supplementary material for: A phase I study of enfortumab vedotin in Japanese patients with locally advanced or metastatic urothelial carcinoma
Source: Invest New Drugs. 2019 Aug 14;38(4):1056–66. doi: 10.1007/s10637-019-00844-x (PMC7340645; doi:10.1007/s10637-019-00844-x)
Supplement: Supplementary file 2 — (PDF 236 kb) [file 10637_2019_844_MOESM2_ESM.pdf]

Online Resource 2

A Phase I Study of Enfortumab Vedotin in Japanese Patients With Locally Advanced or Metastatic Urothelial Carcinoma

Investigational New Drugs

Shunji Takahashi[1], Motohide Uemura[2], Tomokazu Kimura[3], Yoshihide Kawasaki[4], Atsushi Takamoto[5], Akito Yamaguchi[6], Amal Melhem-Bertrandt[7], Elaina M. Gartner[8], Takashi Inoue[9], Rio Akazawa[9], Takeshi Kadokura[9], Toshiki Tanikawa[10]

[1]The Cancer Institute Hospital of Japanese Foundation for Cancer Research, Tokyo, Japan; [2]Osaka University Hospital, Osaka, Japan; [3]University of Tsukuba Hospital, Tsukuba, Japan; [4]Tohoku University Hospital, Sendai, Japan; [5]Okayama University Hospital, Okayama, Japan; [6]Harasanshin Hospital, Fukuoka, Japan; [7]Astellas Pharma Global Development, Northbrook, IL, USA; [8]Seattle Genetics, Seattle, WA, USA; [9]Astellas Pharma, Inc., Tokyo, Japan; [10]Niigata Cancer Center Hospital, Niigata, Japan

Corresponding author:

Shunji Takahashi, MD

E-mail: s.takahashi-chemotherapy@jfcr.or.jp

Defined Kaplan-Meier Plot Censorship Criteria

The following were censorship criteria for duration of response (DoR):

- If patient had neither PD nor death, the DoR was censored at last radiological tumor assessment date on or prior to the cutoff date, or at the date of first confirmed complete response/partial response if no subsequent radiological assessment was available
- If patient took any new anticancer therapy before any PD event or death, the DoR was censored at the date of last tumor assessment date prior to the date of new anticancer therapy

The following were censorship criteria for progression-free survival (PFS):

- If patient had neither PD nor death, the PFS was censored at the last radiological tumor assessment date
- If patient took any new anticancer therapy before any PD event or death, the PFS was censored at the date of the last tumor assessment during study period prior to the date of new anticancer therapy; in the case that patient did not have postbaseline tumor assessment, the PFS was censored at the date of first dose
